# Cartilage development requires the function of Estrogen-related receptor alpha that directly regulates *sox9* expression in zebrafish

**DOI:** 10.1038/srep18011

**Published:** 2015-12-10

**Authors:** Yong-Il Kim, Joon No Lee, Sushil Bhandari, In-Koo Nam, Kyeong-Won Yoo, Se-Jin Kim, Gi-Su Oh, Hyung-Jin Kim, Hong-Seob So, Seong-Kyu Choe, Raekil Park

**Affiliations:** 1 Department of Microbiology and Center for Metabolic Function Regulation, Iksan, Jeonbuk, 570-749, South Korea; 2 Integrated Omics Institute, Wonkwang University School of Medicine, Iksan, Jeonbuk, 570-749, South Korea

## Abstract

Estrogen-related receptor alpha (ESRRa) regulates a number of cellular processes including development of bone and muscles. However, direct evidence regarding its involvement in cartilage development remains elusive. In this report, we establish an *in vivo* role of Esrra in cartilage development during embryogenesis in zebrafish. Gene expression analysis indicates that *esrra* is expressed in developing pharyngeal arches where genes necessary for cartilage development are also expressed. Loss of function analysis shows that knockdown of *esrra* impairs expression of genes including *sox9, col2a1, sox5, sox6, runx2* and *col10a1* thus induces abnormally formed cartilage in pharyngeal arches. Importantly, we identify putative ESRRa binding elements in upstream regions of *sox9* to which ESRRa can directly bind, indicating that Esrra may directly regulate *sox9* expression. Accordingly, ectopic expression of *sox9* rescues defective formation of cartilage induced by the knockdown of *esrra*. Taken together, our results indicate for the first time that ESRRa is essential for cartilage development by regulating *sox9* expression during vertebrate development.

Estrogen-related receptors (ESRRs), an orphan nuclear receptor family, were originally identified due to sequence similarity with estrogen receptors (ERs), and accordingly share many target genes with ERs^1^,^2^,^3^. However, ESRRs are not responsive to estrogen and their ligands are yet to be discovered. Recent studies indicate that members of ESRRs participate in a number of biological processes including metabolism, reproduction, and development^4^. In particular, ESRR alpha (ESRRa) and ESRR gamma (ESRRg) are key metabolic regulators of energy homeostasis, and abnormal functions of these proteins are linked to metabolic syndromes including diabetes and fatty liver disease^5^. The role of ESRRa in cellular metabolism is largely dependent on its transcriptional regulation of mitochondrial function and biogenesis through collaboration with the peroxisome proliferator-activated receptor γ coactivator-1 alpha (PGC1a)^6^. Beside PGC1a/b, ESRRa has been shown to potentiate a metabolic syndrome by acting downstream of mammalian target of rapamycin (mTOR)^7^ and also promotes hypoxic adaptation of cancer cells by stabilizing hypoxia inducible factor 1-alpha (HIF1-a) from degradation^8^. These reports together with other studies solidify the role of ESRRa in maintaining energy homeostasis.

The role of ESRRs during animal development may also be linked to metabolic regulation by which developing embryos meet their high energy demand for growth. A complex expression pattern of ESRRs during animal development seems to be consistent with the potential roles for ESRRa during appropriate developmental programs of tissues and organs in mouse and zebrafish^9^. However, aside from muscle development, the roles of ESRRs in other tissues including bone and cartilage have just begun to be investigated^10^. During bone development, ESRRs are shown to be involved in differentiation and function of osteoblasts and osteoclasts with potential involvement of PGC1 (reviewed in^11^). A potential role for ESRRa in chondrocyte development was largely determined by its ability to regulate expression of *SOX9*, the master chondrogenic regulator, in cell culture studies^12^. A concerted action of ESRRa together with PGC1a for *SOX9* expression in osteoarthritic (OA) chondrocytes also supports the positive involvement of ESRRa in chondrocyte development^13^. However, more direct evidence using an animal model is necessary to demonstrate the *in vivo* role of ESRRa in chondrocyte development.

In this report, we used the zebrafish model to examine the role of *esrra* in cartilage development during vertebrate embryogenesis. Expression of *esrra* is colocalised with genes necessary for cartilage development in pharyngeal arches during zebrafish embryogenesis. Knockdown of *esrra* induces abnormally formed cartilage structure in pharyngeal arches. Importantly, we found conserved ESRRa binding elements in the upstream regions of *sox9* to which ESRRa can directly bind. Accordingly, *sox9* overexpression partially rescues defective formation of cartilage induced by knockdown of *esrra*. These results establish ESRRa as a critical regulator of *sox9* required for cartilage development *in vivo*.

## Results

### Differential activities of Esrra are required for different developmental programs during zebrafish embryogenesis

**Figure 1:**
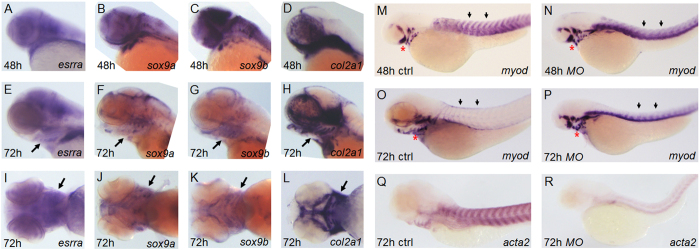
*esrra* is expressed in cartilaginous regions during zebrafish embryogenesis. (**A–L**) Embryos at the indicated stages were subjected to *in situ* hybridization to analyse expression of *esrra*, *sox9a*, *sox9b* and *col2a1*. Arrows in (**E–L**) indicate pharyngeal arches where cartilage development occurs. Note that expression of *esrra* is largely overlapping with that of *sox9a*, *sox9b* and *col2a1.* (**M–R**) Embryos were injected with either *MOctrl* or *MOesrra*, raised to 48 hpf or 72 hpf as indicated, and analysed by *in situ* hybridization for expression of *myod* and *acta2*. *myod* expression in somites (arrows) is drastically decreased as muscle becomes differentiated from 48 hpf to 72 hpf in control embryos (compare **M** and **O**), while it sustains in *MOesrra*-injected embryos (compare **N** with **P**). In contrast, robust expression of *acta2*, a differentiated muscle marker, in control embryos is almost lost in *MOesrra*-injected embryos (compare **Q** with **R**). In addition, expression of *myod* in the pharyngeal arches (red asterisks) does not change from 48 hpf to 72 hpf in *MOesrra*-injected embryos, while *myod* in control embryo is expressed in a different subset of cells at 72 hpf as compared to that in 48 hpf. Embryos are shown in lateral views with anterior to the left except **I**–**L** where embryos are shown in ventral views.

Initially, we examined expression of *esrra* during zebrafish embryogenesis by performing *in situ* hybridization. The transcript initially appears very weakly at 2 hours post fertilization (hpf) and becomes abundant in the posterior region at 10 hpf. Later during development, *esrra* is expressed in various tissues including brain, somites and pronephric duct primordium (Supplementary Fig. 1A–G). The *esrra* expression pattern is consistent with that reported in The Zebrafish Model organism Database (www.zfin.org). To determine whether *esrra* is expressed in cartilaginous elements, we compared its expression with that of *sox9a*/*b* and *col2a1* in developing pharyngeal arches. Expression of *esrra* overlaps with that of *sox9a*/*b* and *col2a1* at both 48 hpf (Fig. 1A–D) and 72 hpf (Fig. 1E–L) when cartilaginous cells are differentiate. This result suggests a potential role for *esrra* in cartilage development in zebrafish. However, a previous knockdown study in zebrafish showed severe gastrulation defects associated with regulatory roles of *esrra* in morphogenetic movement, which precluded further analysis for the role of *esrra* in animal development^14^. Since a gene may not contribute equally to the development of different cell types, we tested whether *esrra* is the case for different developmental programs. Indeed, we found that the degree of *esrra* knockdown matches the phenotypic severity of resulting embryos. For example, morpholinos (*MOs* that interfere with either transcription or splicing of *esrra*) at a lower dose induce a smaller head, shorter body length and curved body axis while *MOs* at a higher dose mimic the gastrulation defect phenotype that was reported previously (Supplementary Fig. 1H–K). Notably, we found that knockdown of *esrra* at a low dose induces defective muscle differentiation, a well-known effect of *esrra* deficiency. In particular, expression of *myod* in somites is decreased at 3 days post fertilization (dpf) in control embryos while retained in 80% (70/88) of *esrra* knockdown embryos (Fig. 1M–P). In addition, we find the expression of another muscle marker gene, *acta2,* being significantly reduced at 3 dpf in 81% (52/64) of *esrra* knockdown embryos (Fig. 1Q,R). These results suggest that *esrra* is required for various developmental programs during zebrafish embryogenesis and that it may exert its role with differential activities.

### Knockdown of *esrra* impairs cartilage development in pharyngeal arches

**Figure 2:**
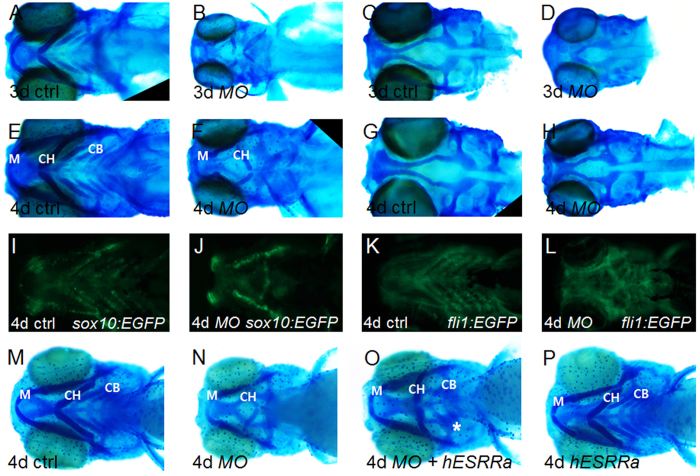
The structure of craniofacial cartilage is disorganised upon knockdown of *esrra*. (**A–H**) Embryos were injected with either *MOctrl* or *MOesrra* and subject to alcian blue staining at 3- or 4 dpf as indicated. Craniofacial cartilage, especially ceratohyal (CH) and ceratobranchial (CB) cartilage, are abnormally developed in *MOesrra*-injected embryos when compared to control embryos. Note that the structure of neural cranium is well organised although smaller in size in *MOesrra*-injected embryos (compare **C** and **G** with **D** and **H**). (**I–K**) *sox10:GPF* (**I,J**) or *fli1:GFP* (**K**,**L**) transgenic embryos were used to confirm the cartilage defects upon knockdown of *MOesrra*. (**M**–**P**) Human *ESRRa* mRNA (*hESRRa*) was co-injected with *MOesrra* into 1-cell stage of embryos which were subject to alcian blue staining. Embryos co-injected with *hESRRa* and *MOesrra* show partial restoration of CB (asterisk in **O**) and repress disorientation of CH induced by *MOesrra* alone (compare **O** with **N**). Misexpression of *hESRRa* at 100 pg does not impair cartilage development (**P**, see the text for detail). Embryos are shown in ventral view with anterior to the left.

With the phenotypic severity corresponding to the degree of *esrra* knockdown, we focused on the role of *esrra* in cartilage development using a low-dose of *MOesrra*. Of note, *esrra* knockdown does not induce significant changes in the expression of other *esrr* members such as *esrrb* and *esrrg* (Supplementary Fig. 1L). Alcian blue staining showed that *esrra* knockdown induces 82% (76/93) of embryos displaying abnormal structure of cartilaginous elements in pharyngeal arches, including a smaller meckels’ arch, reversely-oriented ceratohyal and almost absent ceratobranchial cartilages, as compared to those in control (Fig. 2A,B,E,F). In contrast, the patterning of dorsal neurocranium including ethimoid plate and anterior basicranial commisures is relatively normal in *MOesrra* embryos albeit significantly smaller (Fig. 2C,D,G,H). The defective cartilaginous structure is further verified in two transgenic zebrafish lines, *fli1:EGFP* and *sox10:EGFP* (Fig. 2I–L), by which pharyngeal cartilages can easily be visualised. To confirm that the observed defects in cartilage are specific to *esrra* knockdown, we generated a full-length human *ESRRa* construct and performed a rescue experiment. Since overexpression of *ESRRa* by itself can induce developmentally defective embryos^14^, we used a concentration of *ESRRa* mRNA at which morphological abnormalities can minimally be observed. By examining dosage-dependent phenotypes (Supplementary Fig. 2), we found that *ESRRa* mRNA at 100 pg partly rescues the cartilage defect induced by *esrra* knockdown. In particular, *MOesrra* alone induces 84% (42/50) of embryos having abnormal or missing cartilages, while 68% (54/80) of embryos injected with *MOesrra* together with human *ESRRa* mRNA show ceratobranchial cartilages albeit underdeveloped (Fig. 2M–P). These results indicate that *esrra* is indeed required for cartilage development during vertebrate embryogenesis.

### Knockdown of *esrra* affects development of cranial neural crests that forms pharyngeal arches

**Figure 3:**
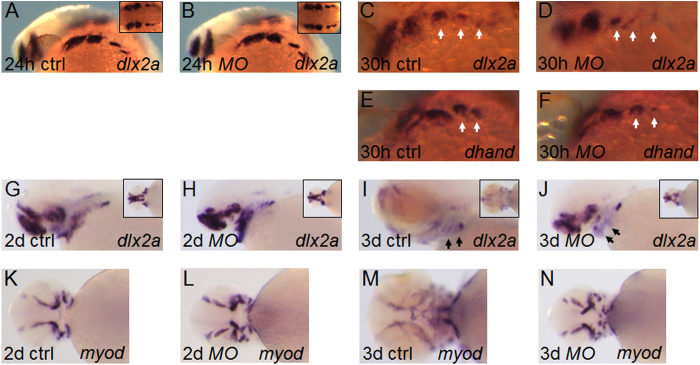
ESRRa regulates development of neural crest cells for cartilage development. (**A–J**) Embryos at 1-cell stage were injected with either *MOctrl* or *MOesrra* and analysed for expression of *dlx2a* or *dhand* by *in situ* hybridization. Expression of *dlx2a* at 24 hpf shows a similar pattern between control and *MOesrra*-injected embryos while expression of both *dlx2a* and *dhand* at 30 hpf shows a slight decrease in the branchial arches (white arrows in **D** and **F**) in *MOesrra*-injected embryos. From 2 dpf to 3 dpf, expression of *dlx2a* becomes largely restricted to pharyngeal cartilage in control embryos (arrows in I), while it is significantly disorganised in the pharyngeal arches of *MOesrra*-injected embryos (arrow in J). (**K–N**) Expression of *myod* from 2 dpf to 3 dpf shows a significant change as the pharyngeal regions in control embryos undergo growth and differentiation (compare **K** to **M**). However, expression of *myod* at 3 dpf remains strikingly similar to that at 2 dpf (compare **L** and **N**), except few additional elements being developed in *MOesrra*-injected embryos. Embryos are shown in either lateral (**A–J**) or ventral views (**K–N**).

Our results suggested that *esrra* knockdown may interfere with specification and/or migration of neural crests that contribute to cartilage elements in pharyngeal arches. We examined *dlx2a* whose expression is found in both premigratory and postmigratory neural crests^11^. Knockdown of *esrra* does not affect *dlx2a* expression at 24 hpf in 100% (63/63) embryos, but slightly reduces it in branchial arches at 30 hpf in 82% (46/56) embryos (Fig. 3A–D). Expression of *dhand* is consistent with that of *dlx2a* at 30 hpf at which it is expressed at a level comparable to control in 1^st^ and 2^nd^ pharyngeal arches but at a slightly reduced level in branchial arches in 84% (37/42) of *MOesrra*-injected embryos (Fig. 3E,F). The strong expression of *dlx2a* in the pharyngeal cartilage in control embryos at 2 dpf, seems to be significantly decreased at 3 dpf when head regions including pharyngeal arches undergo substantial expansion (Fig. 3G,I). In sharp contrast, 80% (45/56) of *MOesrra*-injected embryos continue to express *dlx2a* strongly in the pharyngeal cartilage at 3 dpf in a pattern similar to that found at 2 dpf (compare Fig. 3H–J). Interestingly, *myod* expression in muscular structure of pharyngeal arches in *MOesrra*-injected embryos is reminiscent of *dlx2a* expression. In particular, we find a drastic change in *myod* expression from 2 dpf to 3 dpf in control embryos, while *myod* expression in 80% (70/88) of *MOesrra*-injected embryos at 3 dpf seems to be strikingly similar to that at 2 dpf (Fig. 3K–N). These results suggest that knockdown of *esrra* has a minor role in the specification of cranial neural crests but interferes with growth, maintenance and differentiation of pharyngeal cartilage and other components of pharyngeal arches such as muscles.

### Esrra regulates expression of genes involved in cartilage development

**Figure 4:**
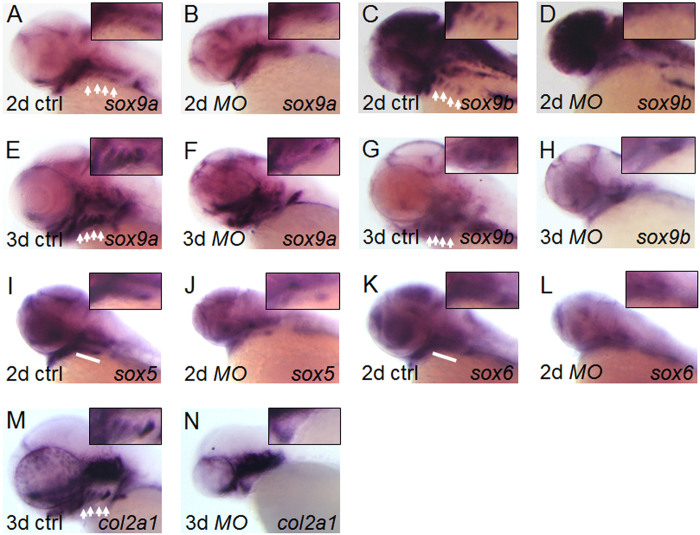
ESRRa regulates expression of genes essential for cartilage development. (**A–N**) Embryos at 1-cell stage were injected with either *MOctrl* or *MOesrra*, raised and analysed at the indicated stages for expression of *sox9a*, *sox9b,sox5, sox6* and *col2a1* by *in situ* hybridization. At 2- and 3 dpf, expression of *sox9* (**A–H**) and *col2a1* (**M** and **N**) is specifically decreased in the branchial arches in *MOesrra*-injected embryos, although it remains comparable in other expression domains as compared to that in control. Expression of *sox5* and *sox6* at 2 dpf is also substantially decreased in *MOesrra*-injected embryos as compared to that in control (**I–L**). White arrows or bars indicate branchial arches and insets shown are magnified views of the branchial regions.

To further test the role of *esrra* in cartilage development, we examined the expression of genes critical for cartilaginous structures in pharyngeal arches. In zebrafish, there are two copies of *sox9* genes, *sox9a* and *sox9b*, both of which cooperate to induce cartilage development^15^. We find that expression of *sox9a* seems to be slightly downregulated in pharyngeal arches as well as in the cranium and somites in 81% (58/72) of *MOesrra*-injected embryos at 1 dpf (Supplementary Fig. 3A,B), although it shows a similar level in the hindbrain at 2 dpf as compared to control embryos. Notably, upon *esrra* knockdown, *sox9a* expression in branchial arches is almost missing or severely decreased at 2 dpf and later in 83% (126/152) of *MOesrra*-injected embryos (Fig. 4A,B,E,F). The reduced expression of *sox9a* in branchial arches correlates well with the loss of cartilaginous elements as shown in Fig. 2. We find that *sox9b* expression is not affected at 1 dpf but is reduced in pharyngeal arches from 2- to 3 dpf in 81% (110/135) of *MOesrra*-injected embryos (Supplementary Fig. 3C,D, and Fig. 4C,D,G,H). This is consistent with a previous report in which expression of *sox9b* in pharyngeal arches only initiates slightly earlier than 48 hpf^15^.

Perturbed expression of *sox9a/b* in pharyngeal arches suggests that chondrocyte differentiation may also be impaired. To examine whether *esrra* regulates chondrocyte differentiation, we examined the expression of *sox5* and *sox6* implicated in chondrogenesis in mammals^16^. Although the role of *sox5* and *sox6* are not clearly demonstrated in zebrafish chondrogenesis, we found that 78% (49/63) and 79% (53/67) of *MOesrra*-injected embryos show significantly downregulated expression of *sox5* and *sox6*, respectively, especially in the branchial arches (Fig. 4I–L). In addition, expression of *col2a1* which encodes a major cartilage matrix component is also severely affected in the pharyngeal arches at 3 dpf in approximately 79% (44/56) of embryos upon knockdown of *esrra* (Fig. 4M,N). These results are consistent with *sox5*, *sox6* and *col2a1* being downstream of *sox9* whose expression is perturbed upon *esrra* knockdown in this study. Furthermore, we also observed expression of other genes important for chondrocyte differentiation and maturation. In zebrafish, *runx2b*, one of the two paralogs of mammalian *runx2,* plays a critical role and regulates *col10a1* expression in both chondrocyte and osteoblast lineages^17^,^18^. We found that both *runx2b* and *col10a1* are significantly reduced upon *esrra* knockdown. In particular, *runx2b* expression in the parasphenoid, ceratobranchials and cleithrum is severely impaired and *col10a1* expression in similar regions is also reduced in 81% (52/64) and 83% (65/78) of embryos, respectively, upon *esrra* knockdown (Supplementary Fig. 3E–H). This result is consistent with a previous report where Sox9b acts upstream of *runx2b* in chondrocyte differentiation and maturation^17^. These results indicate that *esrra* regulates expression of *sox9a/b* and the downstream genes necessary for cartilage development during zebrafish embryogenesis.

### Esrra regulates survival of cartilaginous cells

**Figure 5:**
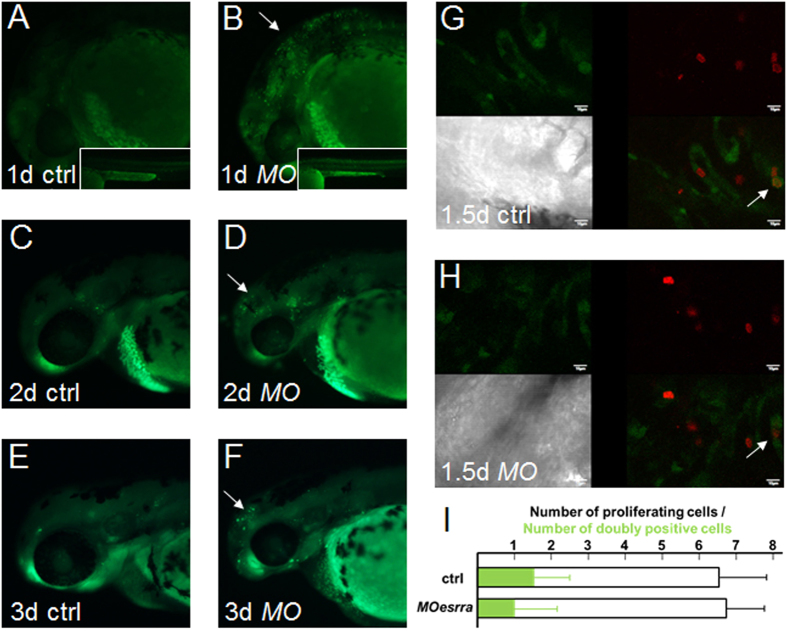
ESRRa regulates survival but not proliferation of cartilaginous cells. (**A–F**) 1-cell stage of embryos were injected with either *MOctrl* or *MOesrra*, raised and subjected to acridine orange stain to determine apoptotic cells at the stages indicated. *Moesrra*-injected embryos display a significant number of apoptotic cells in the head (arrows) and body trunk (insets) at the all observed stages as compared to controls. A combination of *MOesrra* and *MOp53* does not suppress cell apoptosis. (**G,H**) *sox10:GFP* embryos were injected similarly to **A**–**F**, raised to the indicated stages, and processed to determine proliferation of cartilaginous cells by phosphorylated histone H3 immunostaining (pH3, red signal) in pharyngeal regions. White arrows indicate proliferating chondrogenic cells marked by both green and red. Scale bar is 10μm. (**I**) The total number of proliferating cells in the pharyngeal arches is similar between control and *MOesrra*-injected embryos (6.5+/−1.3 vs. 6.8+/−1.0 cells in average, respectively; n = 27). Also, the number of proliferating cartilaginous cells (doubly positive for both green and red) is also similar between control and *MOesrra*-injected embryos (1.5+/−1.0 vs. 1.0+/−1.2 cell in average, respectively; n = 27).

Defective formation of pharyngeal cartilage upon knockdown of *esrra* suggests that cell survival or proliferation of chondrocytes may be dependent on *esrra* activity. Upon knockdown of *esrra*, we find 83% (33/40) embryos displaying an increased number of apoptotic cells in the central nervous system at 1 dpf by acridine orange staining (Fig. 5A,B). In addition, the number of apoptotic cells is also slightly increased at 2- and 3 dpf in 81% (52/64) of *esrra*-knockdown embryos as compared to that in control (Fig. 5C–F), suggesting a repressive role for Esrra in apoptosis. This result may be consistent with a regulatory role for Esrra in the expression of *sox9* which was shown to suppress apoptosis in mammals^16^.

To examine whether knockdown of *esrra* impairs proliferation of pharyngeal chondrocytes, we performed immunostaining using phospho-histone H3 (pH3) antibody that detects nuclei of mitotic cells. We find that comparable numbers of pH3-positive mitotic cells in pharyngeal arches are observed in *esrra*-knockdown embryos as compared to that in control at 36 hpf, 2- and 3 dpf (n > 35 for each stage, Supplementary Fig. 4). To detect proliferating cartilaginous cells more specifically, we utilised *sox10:EGFP* transgenic zebrafish to analyse *sox10*-driven GFP-positive cartilaginous cells that are also pH3-positive. Consistent with Esrra being required for full development of cartilaginous elements in the pharyngeal arches, we find a decreased expression of *sox10*-derived GFP in 79% (62/78) of embryos injected with *MOesrra*. Among the embryos at 1.5 dpf with the reduced GFP expression upon *esrra* knockdown, 82% (27/33) of them have the number of proliferating cartilaginous cells (doubly positive for both GFP and pH3) with the range between 0 and 2 at 1.5 dpf (Fig. 5G–I). Control embryos also contain similar numbers of proliferating cartilaginous cells with average of 1.5, indicating no statistical significance between control and *MOesrra*-injected embryos. These results suggest that Esrra may play a minor role in proliferation but is necessary for survival of cartilaginous cells in the pharyngeal arches.

### Esrra regulates expression of *sox9* whose upstream regions contain putative ESRRa binding elements

**Figure 6:**
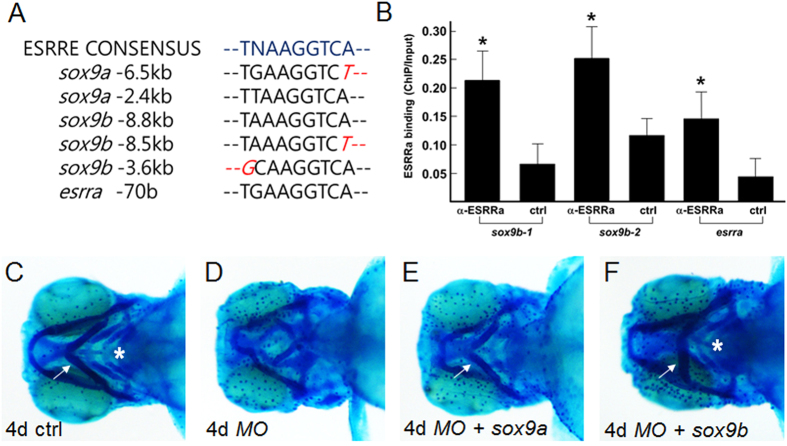
ESRRa directly binds ESRRE consensus elements located upstream of *sox9b*. (**A**) Consensus DNA sequences to where ESRRa is known to bind (ESRRE consensus) are identified upstream of both *sox9a* and *sox9b*. In addition, *esrra* also contains an ESRRE consensus in the proximal promoter region (*esrra* −70 b). (**B**) Embryos at 1-cell stage were injected with *hESRRa* mRNA, raised and collected at 36 hpf for chromatin immunoprecipitation using anti-ESRRa antibody. Significant binding of hESRRa is detected at the ESRRE consensus sites located upstream of *sox9b* (−8.8~−8.5 kb denoted as *sox9b-1*and −3.6 kb as *sox9b-2*), but not detected at the site found upstream of *sox9a* (−6.5 kb). hESRRa also binds to the proximal promoter of *esrra* itself. Statistical analysis of pair-wise samples was performed using IBM SPSS Statistics 22 software. Values with *p* < 0.05 were considered to be statistically significant (indicated by asterisks). (**C–F**) Embryos were injected with *MOesrra* together with *sox9a* or *sox9b* mRNA, and subject to alcian blue staining at 4 dpf. White arrows indicate correctly-oriented ceratohyal cartilage, and white asterisks point to ceratobranchial arches. Note that overexpression of *sox9b* efficiently rescues defective cartilage induced by *esrra* knockdown, while overexpression of *sox9a* only partially rescues proper formation of ceratohyal cartilage.

ESRRa is known to bind a conserved binding sequence and hence regulates the expression of target genes. We found several putative binding elements for ESRRa in the upstream regions of *sox9a* and *sox9b*, and also an element in the proximal promoter of *esrra* itself (Fig. 6A). We tested the possibility that Esrra directly regulates *sox9a* and *sox9b* in zebrafish since *esrra* knockdown decreases expression of both as shown in Fig. 4. To examine the direct association of Esrra to these sites, we performed chromatin immunoprecipitation assay using zebrafish embryos injected with human *ESRRa* mRNA. As shown in Fig. 6B, ESRRa is reliably recruited to two potential ESRRa binding sequences located upstream of *sox9b*. In addition, we also find direct association of ESRRa to a putative binding site located within the proximal promoter of *esrra* gene itself (Fig. 6B). In support of this result, an *in vivo* reporter assay shows robust GFP expression driven by endogenous Esrra to the putative ESRRa binding element located upstream of *sox9b* (−3.6 kb), while a control vector containing only Carp beta-actin minimal promoter and GFP cDNA results in few GFP-positive cells (Supplementary Fig. 5). Importantly, coinjection of the ESRRa binding element of *sox9b* together with *MOesrra* significantly reduces the number of GFP-expressing cells, strongly suggesting that Esrra may directly regulate *sox9b* expression *in vivo*.

Since Esrra may directly regulate expression of *sox9* as well as *esrra* itself, we reasoned that *sox9a/b* overexpression may rescue defective cartilage induced by *esrra* knockdown if Esrra acts through Sox9. To examine whether *sox9* overexpression can rescue defective cartilage in *esrra*-knockdown embryos, we cloned full length cDNAs of *sox9a* and *sox9b*. Since *sox9* overexpression was reported to induce developmental defects due presumably to ectopic upregulation of target genes^19^, we first determined a concentration of *sox9* at which general development is minimally affected but cartilage development can be rescued in combination of *MOesrra* (Supplemental Fig. 2). As shown in Fig. 6C–F, microinjection of *MOesrra* together with either *sox9a* or *sox9b* mRNA into 1 cell stage of zebrafish embryos can rescue at least in part the defective formation of cartilaginous structures indicating that Esrra indeed regulates expression of *sox9* for cartilage development in zebrafish. These results indicate that Esrra directly regulates *sox9b* and *esrra* itself for cartilage development in zebrafish.

## Discussion

A role for ESRRa in chondrogenesis is largely supported by a previous study in which ESRRa was shown to regulate *SOX9* expression *in vitro*^12^. In this study, we show that *esrra* and *sox9a/sox9b/col2a1* are co-expressed in developing chondrocytes during zebrafish embryogenesis which potentiates a role for Esrra in chondrogenesis *in vivo* (Fig. 1). Previously, disruption of *esrra* expression in zebrafish was reported to induce defective gastrulation due to abnormal morphogenetic cell movement^14^, which precluded further analysis for the role of *esrra* in animal development. We re-evaluated the roles of *esrra* in animal development during zebrafish embryogenesis by a morpholino-based knockdown approach. Consistent with a previous report, we find that near-complete blockage of *esrra* expression induces gastrulation defect. However, we also find that a less-complete knockdown of *esrra* induces embryos with smaller head, defective muscle and abnormal cartilage. The target site of our translation-blocking MO overlaps with that in the previous report, and we do not completely understand the phenotypic variance between the previous report and our study. However, the difference could at least in part be due to a threshold effect resulting from the differential degree of gene knockdown as suggested previously^9^. Consistent with this hypothesis, the observed phenotypes in the cartilage as well as muscles can be detected by injecting low dose of MO, while we find a significant developmental delay upon injection of high dose of MO. Therefore, differential activities of Esrra may be involved in the patterning of different tissues by which muscles and cartilage may require full activities of Esrra while migrating cells during gastrulation only require basal activities. In view of this scenario, other members of Esrrs that are expressed during embryogenesis^4^ may not compensate for a more-complete loss of *esrra* that leads to gastrulation defects. In support of this, we found that *esrra* knockdown does not induce changes in expression levels of *esrrb* and *esrrg*. We note that our phenotypic analysis may represent a mild version of *esrra* knockdown since we chose a MO concentration that does not interfere with gastrulation but cartilage formation.

The mechanism by which Esrra acts in chondrocyte development including cell death, proliferation and differentiation, may depend on transcriptional activation of *sox9b*. First, ChIP analysis reveals an association of human ESRRa to two putative ESRRa binding sequences located upstream of *sox9b*, implicating that ESRRa may directly regulate *sox9b* expression. This finding is further supported by an *in vivo* reporter assay by which we show that endogenous Esrra can drive GFP expression by presumably binding to one of the *sox9b* ESRRa binding elements introduced in a reporter vector. Second, we find decreased expression of *sox9b* in response to *esrra* knockdown, while we do not detect an association of ESRRa in a seemingly well-conserved ESRRa binding sequence located upstream of *sox9a*. Since the presence of a positive regulatory loop between *sox9a* and *sox9b* in developing zebrafish has previously been reported^19^, decreased expression of *sox9b* may in turn influence *sox9a* expression in the cartilage regions. Indeed, we observe a reduced expression of *sox9a* in pharyngeal arches upon *esrra* knockdown. However, we cannot rule out the possibility that ESRRa association to *sox9a* gene may be restricted to a small subset of chondrogenic cells, which causes the binding signal to fall below the detection level due to the heterogeneous nature of samples used for ChIP analysis. Third, phenotypes induced by either a *sox9b* mutation or knockdown in zebrafish in previous reports are remarkably similar to those observed in this study upon *esrra* knockdown^19^,^20^. In particular, the observation of embryos with increased cell death and defective cell differentiation upon disruption of either *esrra* or *sox9b* indicates a functional ESRRa-Sox9 axis for supporting a complete set of crest-driven chondrocyte development in pharyngeal arches. Fourth, ectopic expression of *sox9* rescues at least partially *MOesrra*-induced defects in cartilage development. These findings strongly suggest that ESRRa acts through Sox9 activity during cartilage development.

We find a significant sequence homology between mammalian and zebrafish ESRRa, which in turn suggests functional homology between human and zebrafish. We observe that knockdown of *esrra* induces defective muscle development, a well-known phenotype associated ESRRa deficiency in mice. In addition, we show that *MOesrra*-induced phenotype can partially be rescued by ectopic expression of human *ESRRa*. Recently, ESRRa reportedly cooperates with other partner proteins, such as PGC1a/b, HIF1-a and mTOR, in regulating cellular metabolism and hence animal development. Therefore, it will be interesting to understand how ESRRa contributes its function during animal development, a sophisticated process that orchestrates diverse cellular programs into a coordinated series of events necessary for achieving proper animal body plan and function. In summary, we report for the first time a direct involvement of ESRRa in cartilage development *in vivo* achieved at least in part by regulating expression of *sox9*, an essential component of chondrogenesis.

## Methods

### Animal care and transgenic zebrafish

The zebrafish and their embryos were handled and staged according to standard protocols^21^. All experimental protocol was approved by the Committee for Ethics in Animal Experiments of the Wonkwang University (WKU15-102) and carried out under the Guidelines for Animal Experiments.

### Constructs and mRNA or morpholino (MO) microinjection

Human *ESRRa* construct was cloned into pCS2^+^ after digesting out the open reading frame of *ESRRa* from a construct purchased from Addgene, USA. Zebrafish full length *sox9a* and *sox9b* cDNA were cloned into pCS2^+^ and used for mRNA synthesis after linearization. Constructs for making probes for *in situ* hybridization were cloned into Topo vector (*esrra*, *col2a1*, *acta2*, *sox5*, *sox6*, *col10a1*, *sox9a* and *sox9b*) or reported previously (*dlx2a*, *dhand* and *myod*). mMessage mMachine kit (Ambion, USA) was used to synthesize mRNA and 100 pg of mRNA was injected for the rescue experiments. Morpholinos (MOs) were purchased from Gene Tools, OR, USA. 3 ng of MO was used to examine cartilage defects and 10 ng MO was used to confirm gastrulation defects. MO sequences are: 5′-CGTCGTTCTCTGGAAGACATGATAC-3′ (translation-blocking MO) and 5′-ATTGCCTGTTGGATGAAGGGAAACC-3′ (splicing-blocking MO), and 5′- AACATACATCAGTTTAATATATGTA-3′ (control MO). A construct for GFP reporter assay *in vivo* was initially generated by cloning 282 base pairs including a putative ESRRa binding sequence at the upstream of *sox9b* (primers used: F-5′CCTGACCATTACTCAGCGGATGGAGTAT3′ and R- 5′CATCCATGCTCAACTAACCCTCAGCA3′) into pGEM-T easy vector (Promega, USA) in which a DNA fragment encompassing Carp beta-actin minimal promoter and GFP cDNA was subsequently inserted downstream of the ESRRa binding element. All clones were confirmed by DNA sequence analysis. 50 pg of either control construct or GFP reporter alone or in combination with *MOesrra* was injected into 1-cell stage of zebrafish embryos, and live images at various developmental stages were taken using a Leica M165FC microscope equipped with Leica DFC500.

### *In situ* hybridization, immunostaining, acridine orange staining and alcian blue staining

*In situ* hybridization and immunostaining were performed as previously described^22^. Phosphorylated histone H3 antibody was purchased from Santa Cruz Biotechnology, Inc., USA. For acridine orange staining, embryos were manually dechorionated and incubated in acridine orange (0.5μl of 4% acridine orange (Sigma, USA) in 10 ml egg water) for 1 hour at room temperature. Embryos were washed thrice with egg water and then observed using a fluorescence microscope (Leika M165FC). Alcian blue staining was performed as described previously^18^. Briefly, embryos were fixed in 4% paraformaldehyde and maintained in 100% MeOH at −20 °C until use. Embryos were washed several times in PBST (Phosphate buffered saline with 0.1% tween) and bleached in 30% hydrogen peroxide by exposing to bright light for 2 hours. Embryos were rinsed twice with PBST, transferred into alcian blue solution (1% concentrated hydrochloric acid (HCl), 70% ethanol, 0.1% alcian blue (Sigma, USA) and stained overnight. Embryos were washed three times with acidic ethanol (5% concentrated HCl, 70% ethanol) and twice with PBST. Embryos were photographed using Leica M165FC microscope equipped with Leica DFC500 or Olympus IX81, a confocal microscope.

### Reverse transcription (RT)-PCR and western blot

To determine MO efficiency, total RNA was prepared from 10 embryos at 1 dpf using Trizol (Ambion, USA) following manufacturer’s instructions. First strand cDNA was synthesised (Roche, USA) and quantitative PCR was performed. Primer sequences used are F-5′ACTGGTAGTGGAGGAGGGC3′ and R-5′CTCTTGTTGTACTTCTGTCGTC3′. RT-PCR analysis for *esrrb* and *esrrg* was performed using the following primers: F-5′ ATCGGGATACCACTATGGTGTGGCCT3′ and R-5′ GTGAGCCCCAGGTAAGCTGTGTTTT3′ (*esrrb*) and F-5′ AATACGACATCGAGGCCAGTCACATG3′ and R-5′ ATAGTGGTAACCAGAAGCGATGTCCCC3′ (*esrrg*). Western blot analysis was performed as previously described^23^. Anti-ESRRa or -b-actin antibodies were used to specifically detect hESRRa (including endogenous zebrafish Esrra) or b-actin, respectively, after running a gel with embryo lysates equivalent to 10 embryos per lane.

### Chromatin immunoprecipitation

Chromatin immunoprecipitation was performed as described previously^22^ with following modifications. In brief, embryos injected with human *ESRRa* mRNA and raised to 36 hpf were dissociated into single cells and crosslinked in 1% formamide solution. Samples were sonicated with 35% amp for a total of 2 minutes using Epishear Probe Sonicator (Active Motif, USA) and then pre-cleared using 80 μl of protein A agarose slurry containing salmon sperm DNA (Merck Millipore Corp., USA) for 1 hour at 4 °C. Samples were spun and supernatants were divided equally into 2, each labelled as an antibody or a control. 40 ul of protein A agarose slurry was added to both samples and 1 μg anti-ESRRa antibody (Abcam, USA) was added only to the samples labelled as antibody. All the remaining steps were followed as described. Primer sequences for quantitative PCR are: *esrra* (F-5′AAACACCACCTCACCTGCACATATTG3′, and R-5′GTCAGAGCGTCGTT CTCTGGAAG3′), *sox9b-1* (F-5′GTGTGAGATCAGAGTTAATAAAGGTCA3′ and R-5′CCCAGCCAATCACAGTCAGTTAGCA3′), *sox9b-2* (F-5′ CTCCACACAGAAACACCAACTGACCC3′ and R-5′ TACGTGCAGAGTGGCGGCACGGT3′). Statistical analysis was performed using IBM SPSS Statistics 22 software (International Business Machines Corp., USA). Values with *p* < 0.05 (indicated by asterisks) were considered to be statistically significant.

## Additional Information

**How to cite this article**: Kim, Y.-I. *et al.* Cartilage development requires the function of Estrogen-related receptor alpha that directly regulates *sox9* expression in zebrafish. *Sci. Rep.* **5**, 18011; doi: 10.1038/srep18011 (2015).

## Supplementary Material

Supplementary Information
