# Supplementary material for: Cartilage development requires the function of Estrogen-related receptor alpha that directly regulates sox9 expression in zebrafish
Source: Sci Rep. 2015 Dec 10;5:18011. doi: 10.1038/srep18011 (PMC4675082; doi:10.1038/srep18011)

**Supplementary information**

# Cartilage development requires the function of Estrogen related receptor alpha that directly regulates *sox9* expression in zebrafish

Yong-Il Kim[1#], Joon No Lee[1#], Sushil Bhandari[1], In-Koo Nam[1], Kyeong-Won Yoo[1], Se-Jin Kim[1], Gi-Su Oh[1], Hyung-Jin Kim[1], Hong-Seob So[1], Seong-Kyu Choe[1,2,*] and Raekil Park[1,*]

[1]Department of Microbiology and Center for Metabolic Function Regulation, and [2]Integrated Omics Institute, Wonkwang University School of Medicine, Iksan, Jeonbuk, South Korea

# Equal contribution by the first two authors.

* Correspondence: Seong-Kyu Choe and Raekil Park

## Supplementary Figure legends

**Supplementary Figure 1.** Differential activities of ESRRa may be required for different developmental programs. (A-G) Embryos were raised to the indicated stages and analysed for expression of *esrra* by in situ hybridization. *esrra* transcripts are weakly detected as early as 2 hpf, indicating maternal contribution (A). *esrra* is expressed in posterolateral domains of developing embryos at 10 hpf, and becomes ubiquitously expressed including eye, brain, muscle and pronephric duct at 14 hpf and 24 hpf. (H-K) Embryos at 1-cell stage were injected with either *MOctrl* or *MOesrra* and analysed for morphological phenotypes. Embryos injected with *MOesrra* at a high dose (MO-high) show impaired gastrulation (I) as previously reported[14], while those with low doses of *MOesrra* show separable phenotypes (i.e., smaller head, curved body axis and shorter body length) as compared to those in control (compare J with K). (L) Total RNA was extracted from embryos injected with either a low

dose of *MOesrra or MOctrl*, and RT-PCR was performed to determine whether *esrra* knockdown affects expression of *esrra* itself, *esrrb* or *esrrg*. Relative RNA amounts were shown as the percentage of the *b-actin* expression level. Asterisk indicates $p < 0.05$. (M) Schematic drawing shows the structure of *esrra* pre-mRNA. Exons are numbered from 1 to 8, and MO target regions are indicated. Splicing-block *MOesrra* leads to skipping of exon 4, resulting in a truncated protein devoid of a critical Zn-finger domain. Total RNA was extracted from embryos injected with either *MOctrl* or *MOesrra*, and RT-PCR was performed to determine whether *MOesrra* efficiently blocks mRNA maturation (splicing). Note that indistinguishable phenotype is derived by the use of translation-blocking *MOesrra*, suggesting that our MOs specifically knockdown Esrra in zebrafish. M is a molecular-weight size marker. All images were taken by Yong-Il Kim.

**Supplementary Figure 2.** ESRRA activities regulate embryogenesis. (A-C) Human *ESRRa* mRNA was microinjected to 1-cell stage of embryos and morphologies were analysed at 48 hpf. 100 pg *hESRRa* mRNA does not induce morphological defects (B), while 500 pg *hESRRa* induces severe developmental defects including cyclopia (C) as previously reported[14]. (D) Western blot was performed to detect both endogenous and exogenous ESRRa. Note that injection of *MOesrra* decreases the amount of ESRRa. All images were taken by Yong-Il Kim, except D which was taken by In-Koo Nam.

**Supplementary Figure 3. Esrra regulates genes involved in chondrocyte development. (A-D)** At 24 hpf, expression of both sox9a and *sox9b* shows a minimal decrease along the expression domains in *MOesrra*-injected embryos compared to that in control. (E-H)

Expression of *runx2b* (E, F) and *col10a1* (G, H) is significantly reduced upon *esrra* knockdown.; br, branchiostegal ray; cb, ceratobranchial; ch, ceratohyal; cl, cleithrum; hs, hyosymplectic; ma, mandibular; op, operculum; ps, parasphenoid.

**Supplementary Figure 4.** ESRRa may play a minimal role in cell proliferation in pharyngeal arches. (A-J) *sox10:GFP* transgenic embryos at 1-cell stage were injected with either *MOctrl* or *MOesrra*, raised to indicated stages, and analysed for cell proliferation by immunostaining with anti- phosphorylated histone H3(pH3) antibody. GFP-positive cells in pharyngeal arches are persistently reduced at all stages observed, the number of proliferating pH3-positive cells (cells in red) in *MOesrra*-injected embryos is similar to that in control. Embryos are shown in lateral views (A-D'and G-H') or ventral views (E-F' and I-J'). All images were taken by Yong-Il Kim.

**Supplementary Figure 5.** Esrra may directly regulate *sox9* expression *in vivo.* (A-D) *in vivo* reporter assay was performed to visualise GFP expression based on the binding ability of endogenous Esrra to the putative ESRRa binding element located upstream of *sox9b* (-3.6kb). A reporter construct contains 282 base pairs including the ESRRa binding element upstream of *sox9b,* Carp beta-actin minimal promoter and GFP cDNA in pGEM-T easy vector. A control vector that only contains Carp beta-actin minimal promoter and GFP cDNA was also generated. The resulting constructs were individually microinjected to examine whether endogenous Esrra can drive GFP expression *in vivo*. We found that the construct containing the ESRRa binding element upstream of *sox9b* gives rise to GFP expression in a number of cells where endogenous Esrra is expressed (B). The scattered pattern of GFP expression may be attributed to the nature of DNA injection which does not uniformly distribute in embryonic

cells. In contrast, coinjection of the ESRRa binding element together with *MOesrra* significantly reduces the number of GFP-expressing cells (C). Furthermore, injection of the control construct results in few GFP-positive cells (D), supporting our result from chromatin immunoprecipitation. A construct that contains the ESRRa binding element upstream of *sox9a* (-2.4kb) also drives GFP expression (E), suggesting that the ESRRa binding element may also operate *in vivo*.

Supplementary Figure 1.

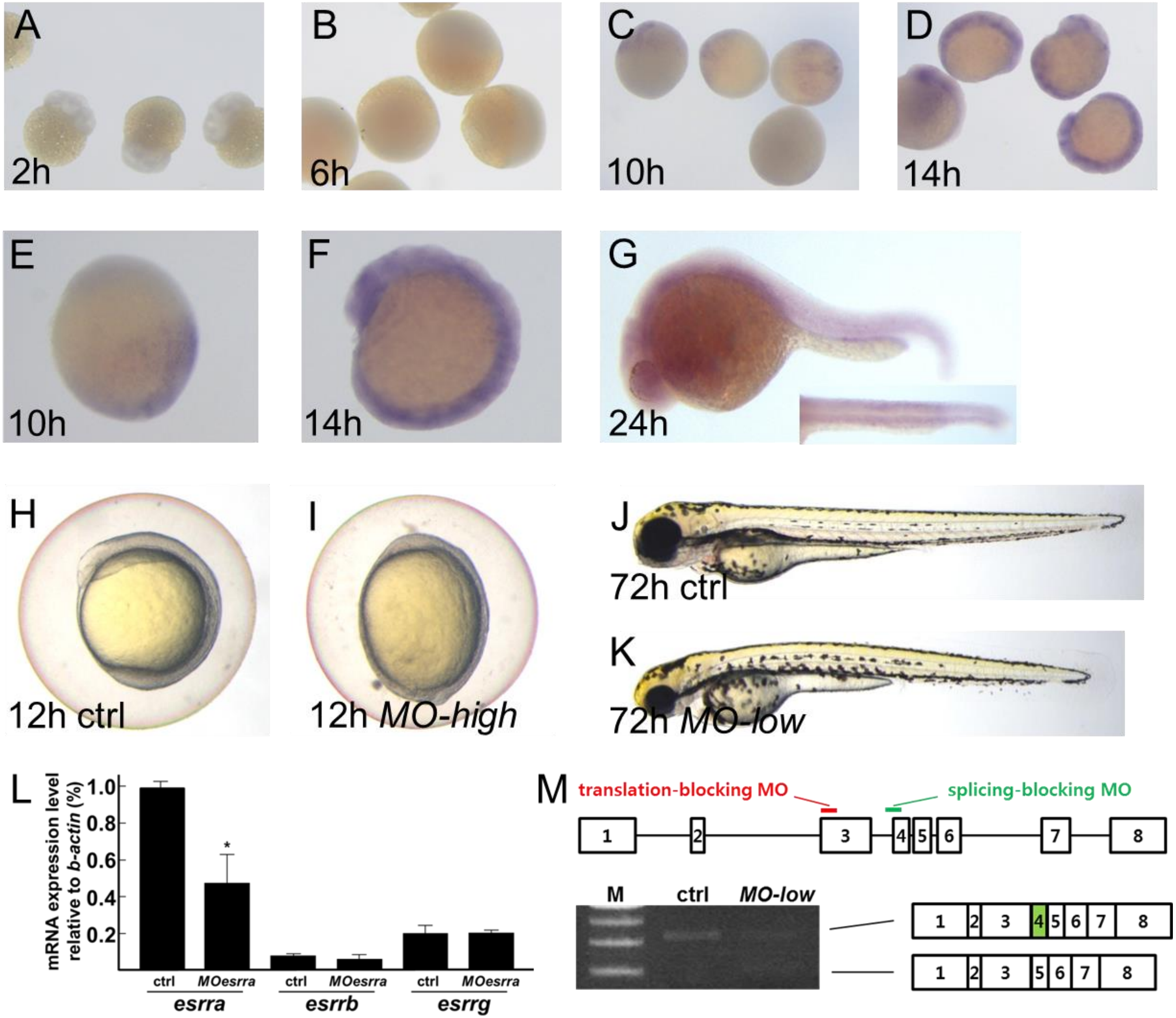

Supplementary Figure 2.

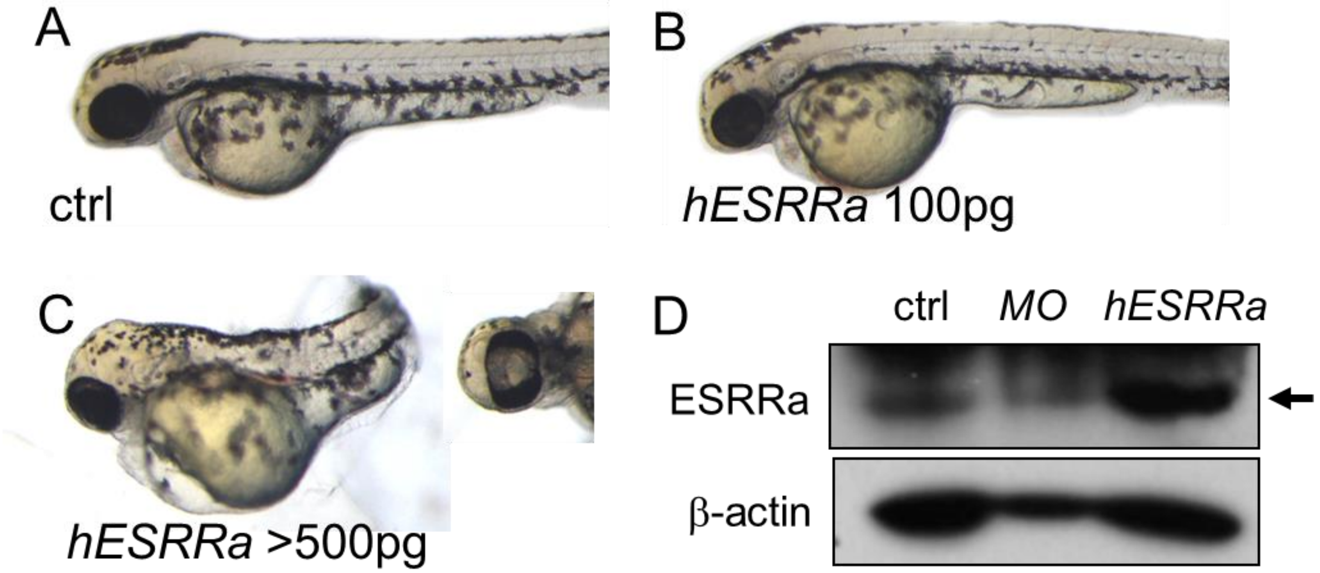

Supplementary Figure 3.

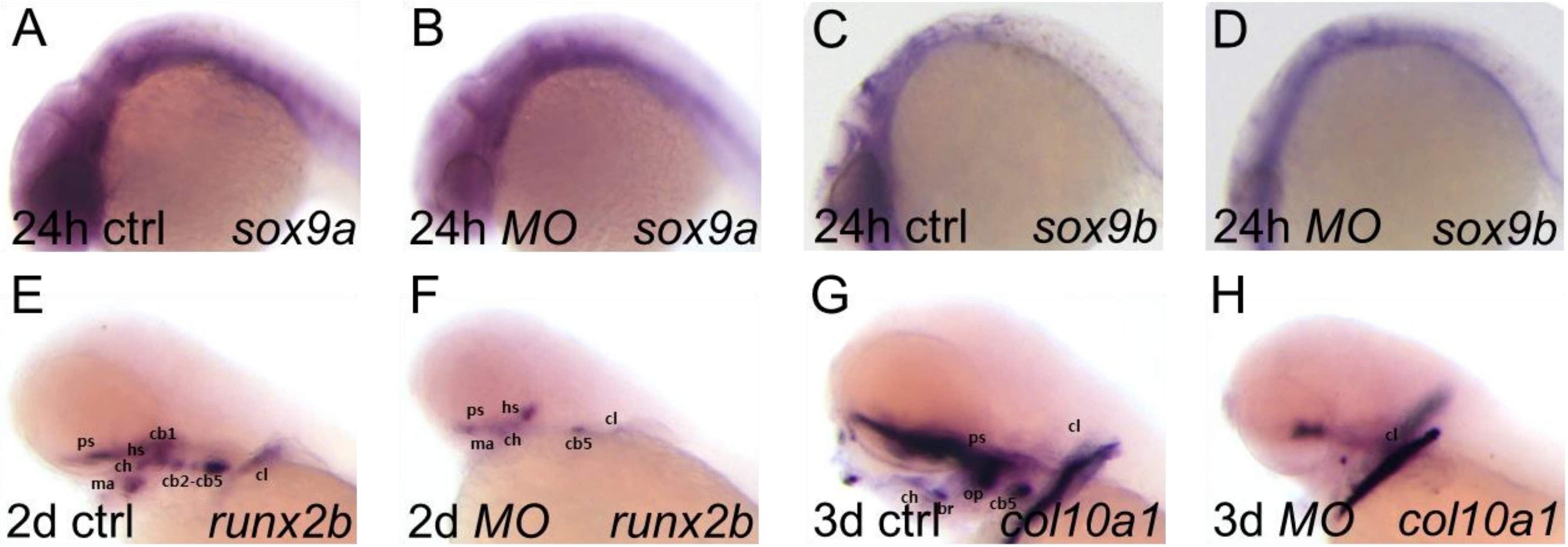

Supplementary Figure 4.

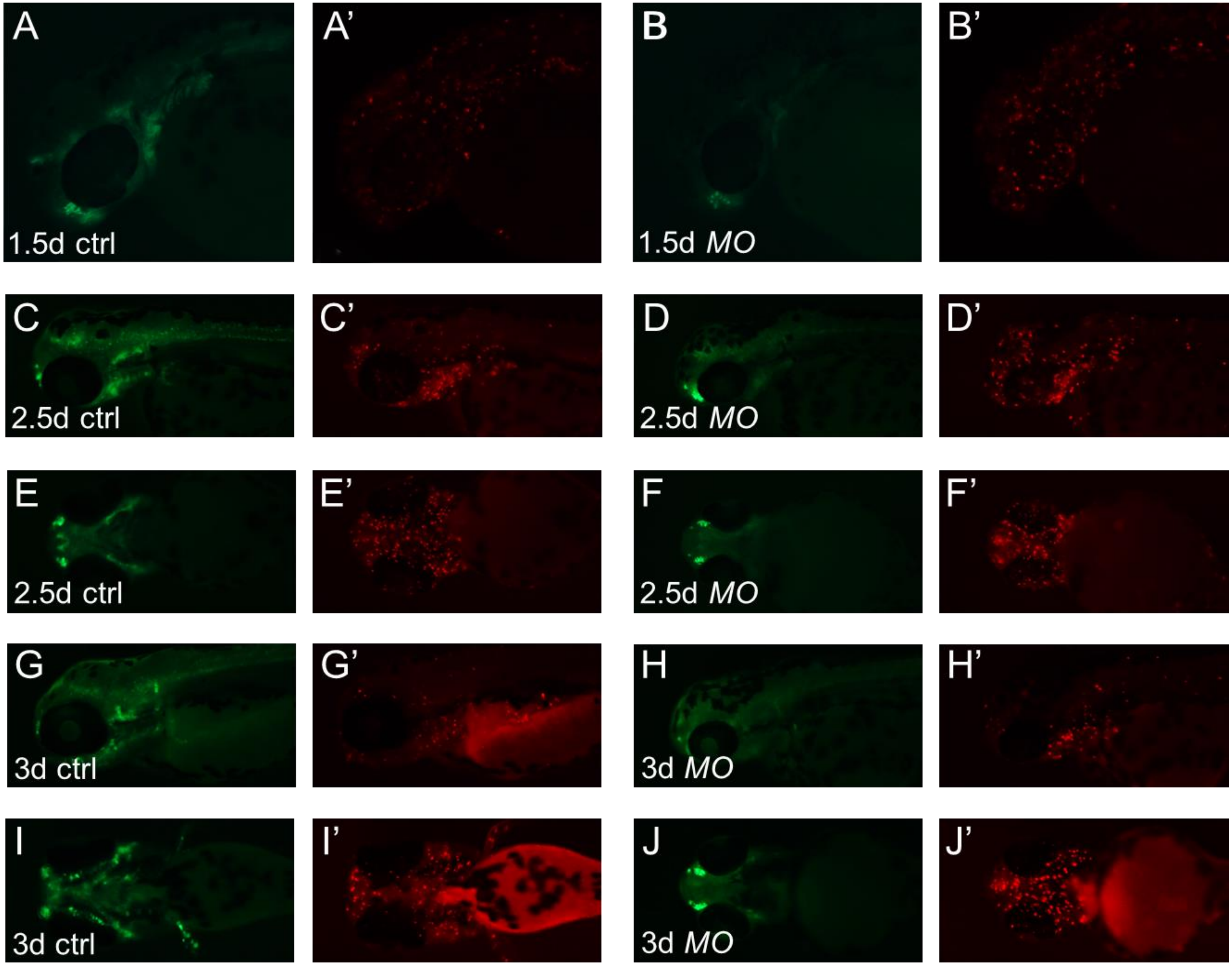

Supplementary Figure 5.

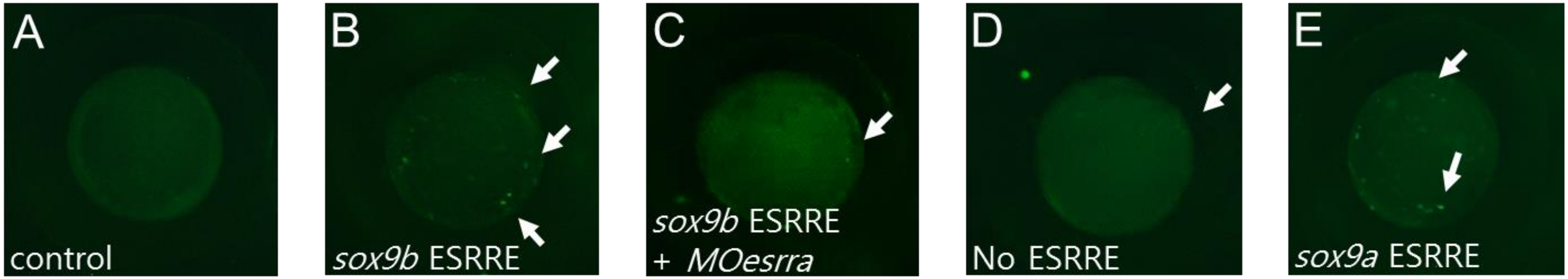

Supplement: Supplementary Information [file srep18011-s1.pdf]
